# Phosphorylation of the ErbB3 binding protein Ebp1 by p21-activated kinase 1 in breast cancer cells

**DOI:** 10.1038/sj.bjc.6604261

**Published:** 2008-02-19

**Authors:** D Akinmade, A H Talukder, Y Zhang, W-m Luo, R Kumar, A W Hamburger

**Affiliations:** 1 Department of Pathology, University of Maryland, Baltimore, MD, USA; 2 Greenebaum Cancer Center, University of Maryland, Baltimore, MD, USA; 3 Molecular and Cellular Oncology, University of Texas MD Anderson Cancer Center, Houston, TX, USA

**Keywords:** Ebp1, PA2G4, phosphorylation, PAK1, breast cancer

## Abstract

The ErbB3 binding protein (Ebp1) is a transcriptional corepressor that inhibits the activity of proliferation-associated genes and the growth of human breast cancer cell lines. Treatment of breast cancer cells with the ErbB3 ligand heregulin (HRG) results in increased phosphorylation of Ebp1 and transcriptional repression. The p21-activated serine/threonine kinase 1 (PAK1), which plays an important role in breast cancer progression and resistance to the anti-oestrogen tamoxifen, is also activated by HRG. We therefore examined the ability of PAK1 to phosphorylate and regulate the function of Ebp1. We found that PAK1 phosphorylated Ebp1 *in vitro* and mapped the phosphorylation site to threonine 261. Both HRG treatment and expression of a constitutively activated PAK1 in MCF-7 breast cancer cells enhanced threonine phosphorylation of Ebp1. In MCF-7 cells, ectopically expressed Ebp1 bound endogenous PAK1 and this association was enhanced by treatment with HRG. Mutation of the PAK1 phosphorylation site to glutamic acid, mimicking a phosphorylated state, completely abrogated the ability of Ebp1 to repress transcription, inhibit growth of breast cancer cell lines and contribute to tamoxifen sensitivity. These studies demonstrate for the first time that Ebp1 is a substrate of PAK1 and the importance of the PAK1 phosphorylation site for the functional activity of Ebp1 in breast cancer cells.

The ErbB family of tyrosine kinase receptors regulates the growth, differentiation and survival of human breast epithelial cells (Holbro *et al*, 2003b). The EGFR family members include EGFR, also known as the human EGF receptor 1 (HER1, ErbB1), HER2 (ErbB2), HER3 (ErbB3) and HER4 (ErbB4). Ligand binding to ErbB receptors results in the formation of homo- or heterodimers leading to tyrosine phosphorylation of the cytoplasmic C-terminal domains, which provide docking sites for effectors of intracellular signalling (Yarden, 2001). ErbB3 is the only member of the ErbB receptor family that lacks tyrosine kinase activity due to amino-acid substitutions in the conserved kinase domain (Kraus *et al*, 1989). Over a dozen peptides activate the ErbB family, including ligands of the EGF class that bind EGFR and ErbB4 and the heregulin (HRG/NRG) family that bind to ErbB3/4 receptors. The ErbB2 receptor has no known soluble ligand (Holbro *et al*, 2003b).

A wealth of clinical data demonstrates the aberrant expression of ErbB family members in breast cancer (Holbro *et al*, 2003b; Hynes and Lane, 2005). The *ErbB2* gene is amplified in 20–30% of breast carcinomas contributing to more aggressive disease (Slamon *et al*, 1989). The overexpression of ErbB2 has been successfully exploited therapeutically by use of the monoclonal antibody Trastuzumab and tyrosine kinase inhibitors. EGFR is overexpressed in 40% of primary breast cancers and preclinical studies demonstrated the efficacy of the EGFR inhibitor gefitinib in ER-positive EGFR overexpressing tumours. However, clinical efficacy is still being evaluated (Johnston, 2006). ErbB3 overexpression has been noted in breast cancer for some time (Lemoine *et al*, 1992), but the aetiologic and prognostic role of ErbB3 in breast carcinogenesis has only recently been widely recognised. Coexpression of ErbB2 and ErbB3 is significantly associated with decreased patient survival (Wiseman *et al*, 2005). The ErbB2/ErbB3 receptor pair forms the most potent mitogenic receptor complex *in vitro* (Pinkas-Kramarski *et al*, 1996b) and is key to the proliferation of human breast cancer cells (Holbro *et al*, 2003a). The importance of ErbB3 expression in breast cancer has been recently highlighted by the demonstration that continued oncogenic signalling through ErbB3 in human breast cancer cell lines results in the failure of gefitinib to completely inhibit the kinase activity of ErbB2 (Sergina *et al*, 2007).

The lack of an active tyrosine kinase domain necessitates the interaction of ErbB3 with other proteins to exert its biological effects. For example, ErbB2 heterodimerizes with ErbB3 after HRG stimulation, leading to phosphorylation and activation of downstream substrates (Pinkas-Kramarski *et al*, 1996a). The RING finger E3 ubiquitin ligase neuregulin receptor degradation protein-1 associates with ErbB3 in an activation-independent manner and is involved in ErbB3 trafficking or localisation (Diamonti *et al*, 2002; Qiu and Goldberg, 2002). Another ErbB3 binding protein (Ebp1) was isolated in our laboratory during a yeast two-hybrid screen (Yoo *et al*, 2000). ErbB3 binding protein binds ErbB3 in human breast cancer cells (Yoo *et al*, 2000; Ahn *et al*, 2006), but dissociates from the receptor in response to HRG (Yoo *et al*, 2000). Overexpression of Ebp1 inhibits the transcription of reporter genes controlled by Cyclin D1, Cyclin E and c-myc promoters and the transcription of endogenous E2F1 and c-myc genes via its binding to an E2F1 consensus element (Xia *et al*, 2001; Zhang *et al*, 2003; Zhang and Hamburger, 2004). The interaction of Ebp1 with histone deacetylase 2 (HDAC2), Rb and Sin3A is needed for Ebp1 to repress transcription (Zhang *et al*, 2003, 2005; Zhang and Hamburger, 2004). Heregulin, under conditions associated with growth arrest, increases binding of Ebp1 to the E2F1 promoter complex and enhances Ebp1-mediated repression of E2F1-regulated gene transcription (Zhang and Hamburger, 2004). Overexpression of Ebp1 in breast cancer cells inhibits cell growth, while promoting G2/M cell cycle arrest and cellular differentiation (Lessor *et al*, 2000). Previous work from our laboratory has demonstrated, via orthophosphate labelling of whole cells, that Ebp1 is a phosphoprotein. Ser and Thr residues are basally phosphorylated, and this phosphorylation is increased in response to HRG (Lessor and Hamburger, 2001). Specific phosphorylation sites include Ser 360 (Liu *et al*, 2006) and S363 (Akinmade *et al*, 2007) that are important in Ebp1 function. Phosphorylation of Ebp1 at Ser 360 is needed for proper nuclear localisation, and for binding ErbB3 and nuclear AKT (Ahn *et al*, 2006). Phosphorylation at Ser 363 is needed for Ebp1 to bind Sin3A and HDAC2, resulting in repression of Cyclin D1- and Cyclin E-regulated promoters (Akinmade *et al*, 2007).

P21-activated kinase 1 (PAK1), a member of the yeast sterile 20 (Ste20) family of protein kinases (Kumar and Vadlamudi, 2002), is also regulated by HRG and involved in breast cancer progression. P21-activated kinase 1 was first demonstrated to be autophosphorylated via directly binding to the Rho GTPases, cdc42 and rac (Adam *et al*, 1998). Lipids such as sphingosine also activate 3-phosphoinositide-dependent kinase-1, which then directly phosphorylates and activates PAK1 (King *et al*, 2000). Heregulin also indirectly activates PAK1 via the PI3K/AKT pathway (Adam *et al*, 1998). Recent data indicate an important role for PAK1 in breast cancer progression. Activation of PAK1 results in actin phosphorylation in breast cancer cells, leading to a reorganisation of the cytoskeleton that favours cell migration and invasiveness (Adam *et al*, 1998). Activated PAK1 also phosphorylates and activates oestrogen receptor-*α* (ER*α*) independently of ligand stimulation, leading to increased Cyclin D1 expression and cell cycle progression (Holm *et al*, 2006). Mammary glands from catalytically active PAK1 transgenic mice exhibit hyperplasia (Balasenthil *et al*, 2004). Most recently, an association between PAK1 expression and resistance to the anti-oestrogen tamoxifen has been demonstrated. Activation of PAK1 inhibits tamoxifen action *in vitro* and in animal models (Rayala *et al*, 2006). Clinically, the overexpression and nuclear localisation of PAK1 are associated with tamoxifen resistance in a subset of ER-positive tumours (Holm *et al*, 2006).

In light of the potential role of both PAK1 and Ebp1 in breast cancer progression and their activation by HRG, we determined if Ebp1 was a substrate for PAK1. We show here that Ebp1 was phosphorylated by PAK1 on Thr 261 *in vitro*. We found that ectopically expressed Ebp1 bound PAK1 in MCF-7 cells and that this binding was increased by HRG treatment. Mutation of the Thr 261 phosphorylation site significantly affected the biological activity of Ebp1 in breast cancer cell lines.

## MATERIALS AND METHODS

### Cell culture and transfections

MCF-7 and AU565 cells were obtained from the American Type Culture Collection (Manassas, VA, USA) and maintained at 37°C in a humidified atmosphere of 5% CO_2_ in air in RPMI 1640 (Biofluids, Rockville, MD, USA) supplemented with 10% foetal bovine serum (FBS, Sigma, St Louis, MO, USA) and 1% penicillin/streptomycin (P/S). Cells were transfected using Optimem I media (Invitrogen, Carlsbad, CA, USA) and the Fugene 6 mammalian transfection reagent (Roche, Indianapolis, IN, USA) according to the manufacturer's instructions.

### Reagents

Heregulin *β*1 (HRG*β*1) was obtained from R&D Systems Inc. (Minneapolis, MN, USA) and Geneticin (G418) from Invitrogen.

### Plasmids

The glutathione *S*-transferase (GST)-Ebp1 full-length and deletion constructs and their purification have been described previously (Zhang *et al*, 2002). All PAK1 site mutations were created in this vector by site-directed mutagenesis using Stratagene's QuikChange II XL Site-Directed Mutagenesis Kit (La Jolla, CA, USA). The S252A, T261A, S335A and S375A mutations were created in GST-Ebp1 using the following forward primers with sequences starting from the 5′ end:

S252A: CAAACGAGACCCGCTAAACAGTATGGACTG
T261A: CAGTATGGACTGAAAATGAAAGCTTCACGTGCCTTCTTCAGTGAG
S335A: CATGCGGATAACCGCTGGTCCCTTCGAG
S375A: GAAAAAAAAGAAGGCCGCCAAGACTGCAGAGAATG

The CMV10-Ebp1 p52 and GFP-Ebp1 p52 plasmids, encoding Ebp1 translated from the first ATG initiation site (1–394), have been described previously (Akinmade *et al*, 2007). T261A and T261E mutations were created in GFP-Ebp1 using the following forward primers with sequences starting from the 5′ end:

T261A: CAGTATGGACTGAAAATGAAAGCTTCACGTGCCTTCTTCAGTGAG
T261E: CAGTATGGACTGAAAATGAAAGAGTCACGTGCCTTCTTCAGTGAG

All forward primers were reverse complemented to get the reverse primers and were synthesised at the Biopolymer Core Lab at University of Maryland, Baltimore (BCL-UMB). All mutations were verified by automated sequencing in the University of Maryland Biopolymer Core Laboratory. CMV6M-PAK1 (wild type) and the PAK1 mutant (T423E) were a gift of Dr Jonathan Chernoff, Fox Chase Cancer Center (Philadelphia, PA, USA).

### P21-activated kinase 1 assay

*In vitro* kinase assays were performed as described previously (Barnes *et al*, 2003b). Briefly, GST-tagged Ebp1 proteins were used as substrates. The reaction was set up in HEPES buffer (50 mM HEPES, 10 mM MgCl_2_, 2 mM MnCl_2_, 0.2 mM DTT) containing 100 ng of purified human GST-PAK1 enzyme (Alexis Biochemicals, San Diego, CA, USA), 10 *μ*Ci of [*γ*-^32^P] ATP and 25 mM cold ATP. Glutathione *S*-transferase-tagged proteins were resolved by SDS–PAGE and phosphorylated proteins detected by autoradiography.

### Western blot assay

Total cell lysates were prepared by direct lysis with NTEN buffer (20 mM Tris-HCl pH 8.0, 150 mM NaCl, 1 mM EDTA, 0.5% NP-40, 10% glycerol). Protein concentrations were determined using the Biorad detergent compatible Protein Assay Kit. The samples were mixed with Laemmli sample buffer and resolved by SDS–PAGE. Proteins were transferred to PVDF membranes and immunoblotted with the appropriate primary and secondary antibodies. An ECL detection kit (Pierce, Rockford, IL, USA) was used to visualise the bands.

### Antibodies

Primary antibodies included those directed against Ebp1 (rabbit, Upstate, Temecula, CA, USA), Flag M2 (mouse, Sigma), GFP (mouse, Clontech, Mountain View, CA, USA), PAK1 (rabbit, Cell Signaling, Beverly, MA, USA), phosphothreonine (Zymed, Carlsbad, CA, USA) and Actin (rabbit, Sigma). Secondary antibodies included goat anti-rabbit HRP (Biorad, Richmond, CA, USA), goat anti-rat HRP (KPL, Gaithersburg, MD, USA) and sheep anti-mouse HRP (Amersham, Piscataway, NJ, USA).

### Immunoprecipitation

Flag-Ebp1 was immunoprecipitated from MCF-7 cell lysates using anti-Flag M2 Agarose beads (Sigma) as described previously (Xia *et al*, 2001). GFP-Ebp1 was immunoprecipitated from cell lysates using anti-GFP Agarose beads (Medical & Biological Laboratories (MBL), Woburn, MA, USA). The immunoprecipitated proteins were resolved by SDS–PAGE and analysed by western blotting.

### Immunofluorescence

MCF-7 cells stably transfected with GFP-C1 control, GFP-*Ebp1* or GFP-*Ebp1* mutants were visualised using a Carl Zeiss Axiovert 200 microscope and images were captured using the attached digital AxioCam HR and analysed with the AxioVision digital imaging software.

### Dual luciferase assay

A total of 5 × 10^4^ MCF-7 cells per well were transfected with 0.5 *μ*g of GFP, GFP-*Ebp1*, GFP-*Ebp1* T261A or GFP-*Ebp1* T261E plasmids, 0.5 *μ*g of pE2F1-luc (a firefly luciferase reporter gene under the control of the −225 to +1 region of the E2F1 promoter) (Johnson *et al*, 1994), or a Cyclin D1-luc reporter (1–163 of the Cyclin D1 promoter) (Xia *et al*, 2001) and 5 ng of pRL-TK vector (a *Renilla* luciferase reporter gene under the control of the thymidine kinase promoter) using Fugene 6 (Boerhringer Mannheim). Forty-eight hours after transfection, cells were lysed and luciferase activity determined using a dual-luciferase reporter assay (Promega, Madison, WI, USA). The activities of Renilla luciferase were used to normalise any variations in transfection efficiency.

### Colony inhibition assays

Cells were seeded into 12-well plates at 1 × 10^4^ cells per well and cultured in complete media. Cells were transfected with 2 *μ*g of GFP, GFP-*Ebp1*, GFP-*Ebp1* T261A or GFP-Ebp1 T2*61E* plasmids using Fugene 6. After 3 weeks of selection with G418 (800 *μ*g ml^−1^), the plates were stained with crystal violet and the number of surviving colonies was counted.

### Creation of stably transfected cell lines

To establish *ebp1*-overexpressing stable transfectants, subconfluent MCF-7 cells in 100-mm tissue culture dishes were transfected with 10 *μ*g of GFP-tagged wild-type *ebp1* or the T261 mutant plasmids using Fugene-6 according to the manufacturer's protocol. Cells were selected in G418 (800 *μ*g ml^−1^) for 4 weeks and mass cultures obtained.

For creation of *ebp1*-silenced MCF-7 cell lines, siRNA targeted against the coding region beginning at nucleotide 476 (Genbank accession number U87954) (AAGCGACCAGGAUUAUAUUCU) was cloned into the pRNAT-U6.1 lentiviral vector (GenScript Corp., Scotch Plains, NJ, USA). A synthetic oligo encoding this sequence was previously demonstrated to decrease Ebp1 expression in prostate cancer cell lines (Zhang and Hamburger, 2005). Lentiviral particles were prepared using the Invitrogen ViraPower™ system in 293FT cells as described by the manufacturer. MCF-7 cells were transduced with lentiviral stock and polybrene (6 *μ*g ml^−1^) and mass cultures were selected in G418 (800 *μ*g ml^−1^).

### Proliferation assays

For studies assessing the effect of tamoxifen on cell growth, cells (5 × 10^3^) were plated in 96-well plates in complete media. Media were replaced 24 h later with complete media containing the indicated concentrations of tamoxifen (Sigma). Relative cell numbers were determined using a Promega Proliferation Reagent as per manufacturer's instructions with absorbance being read at 490 nm using a Dynex plate reader.

### Statistical analysis

Data were analysed using a two-tailed Student's *t*-test using Microsoft Excel. Differences with a *P*<0.05 were deemed significant.

## RESULTS

### ErbB3 binding protein is a substrate of PAK1

**Figure 1:**
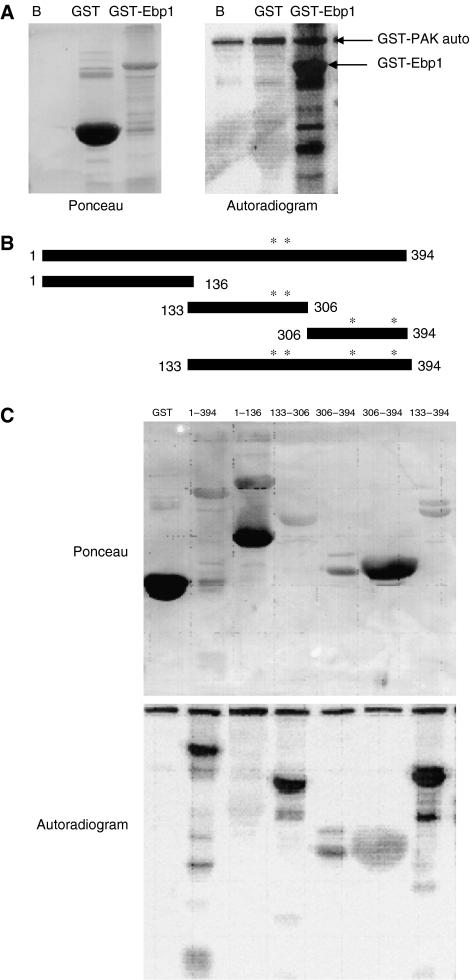
P21-activated kinase 1 phosphorylates Ebp1 *in vitro*. (**A**) An *in vitro* kinase reaction was performed using recombinant GST-PAK1 enzyme and GST or GST-Ebp1 as substrates as described in the Materials and methods. Cell lysates were captured on glutathione agarose beads and phosphorylation of Ebp1 was analysed by SDS–PAGE followed by autoradiography (right panel). The expression of the substrates was analysed by Ponceau staining (left panel). B, beads incubated with enzyme but no substrate. A representative of two experiments is shown. (**B**) Diagram of Ebp1 portion of GST-Ebp1 fusion proteins. The predicted PAK1 phosphorylation sites are indicated by the asterisks. (**C**) The indicated GST-Ebp1 deletion constructs were used as substrates in an *in vitro* PAK1 kinase assay as described in (**A**). A representative of two experiments is shown.

As both Ebp1 and PAK1 are activated by HRG, we tested if PAK1 could phosphorylate Ebp1 *in vitro*. We scanned the Ebp1 sequence for the PAK1 motif (K/R-K/X-X-S/T) and found four putative PAK1 phosphorylation sites at S252, T261, S335 and S375. We first tested the ability of PAK1 to phosphorylate wild-type GST-Ebp1 and Ebp1 deletion constructs. Purified PAK1 enzyme phosphorylated full-length GST-Ebp1 and was autophosphorylated as reported (Barnes *et al*, 2003a) (Figure 1A). We next used GST-Ebp1 deletion constructs (Figure 1B) to further determine the site of PAK1 phosphorylation. One construct encoding amino acids 1–136 did not contain any predicted sites. Another construct encoding amino acids 133–306 contained the S252 and T261 sites. The GST-Ebp1 306–394 construct contained the S335 and S375 sites. Use of these Ebp1 deletion constructs indicated that the Ebp1 phosphorylation site(s) was located between amino acids 133–306 (Figure 1C) in keeping with two of the predicted PAK1 phosphorylation sites.

**Figure 2:**
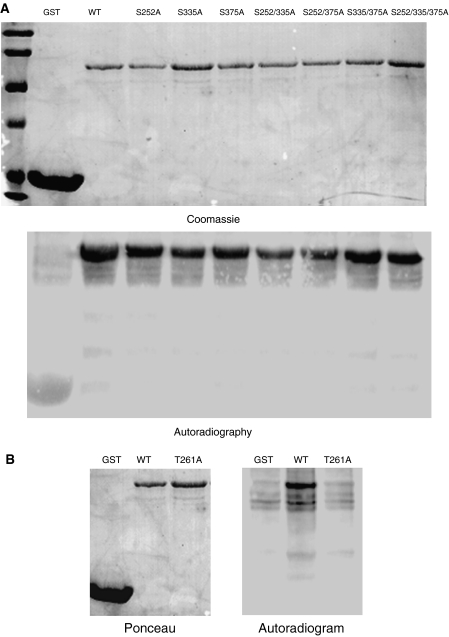
P21-activated kinase 1 phosphorylates Ebp1 at Thr 261. (**A**) Single- and multisite mutations of putative Ser PAK1 sites (S252, S335 and S375) were created in wild-type GST-Ebp1. The resulting mutant proteins were immobilised on glutathione agarose beads and resolved by SDS–PAGE. Gels were stained with Coomassie Blue (top panel). The GST proteins were used as substrates in a PAK1 kinase assay as described in the Materials and methods. Phosphorylation of Ebp1 was analysed by SDS–PAGE followed by autoradiography. Representative of two experiments. (**B**) Mutation of GST-Ebp1 at Thr 261 was created in wild-type GST-Ebp1. The GST proteins were used as substrates in a PAK1 kinase assay as described in the Materials and methods. Phosphorylation of Ebp1 was analysed by SDS–PAGE followed by autoradiography. Representative of two experiments.

To identify specific PAK1 phosphorylation sites in Ebp1, we created single- and double-site mutations of the predicted PAK1 sites in wild-type GST-Ebp1. The resulting mutants were immobilised on glutathione agarose beads. The samples were resolved by SDS–PAGE and Coomassie stained to ensure correct expression (Figure 2A, top). Mutation of putative PAK1 serine phosphorylation sites did not prevent PAK1 phosphorylation (Figure 2A, bottom). In contrast, a single point mutation of Thr 261 to Ala completely abolished PAK1 phosphorylation of Ebp1 *in vitro* (Figure 2B).

**Figure 3:**
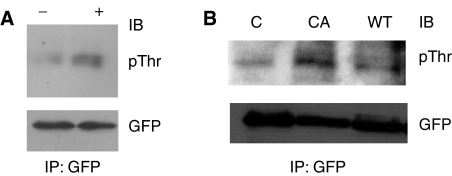
*In vivo* phosphorylation of Ebp1 at Thr residues. (**A**) Thr phosphorylation in response to HRG. MCF-7 cells stably transfected with GFP-*ebp1* were treated with HRG (20 ng ml^−1^) for 10 min (+) or left untreated (−). ErbB3 binding protein was immunoprecipitated with GFP-agarose beads prior to separation by SDS–PAGE. Proteins were transferred to PVDF membranes, and immunoblotted with antibodies to phosphothreonine (pThr) or GFP as indicated. Representative of two independent experiments. (**B**) Constitutively activated PAK1 induces Ebp1 Thr phosphorylation, MCF-7 cells stably transfected with GFP-*ebp1* were transiently transfected with a control plasmid (C), a constitutively activated PAK1 (T423E) or a wild-type PAK1 (WT). Two days after transfection, cells were harvested and immunoprecipitated with GFP-agarose beads. Immunoprecipitated proteins were transferred to PVDF membranes, and immunoblotted with antibodies to phosphothreonine (pThr) or GFP as indicated. Representative of two independent experiments.

We next tested if treatment with HRG, a physiological activator of PAK1, could enhance phosphorylation of Ebp1 at Thr residues *in vivo*. MCF-7 cells stably transfected with GFP-*ebp1* were treated with HRG and then immunoprecipitated with GFP and probed with an anti-Thr antibody. ErbB3 binding protein Ebp1 was basally phosphorylated at Thr and Thr phosphorylation was increased after HRG treatment (Figure 3A) in keeping with previously published data on endogenous Ebp1 in AU565 cells (Lessor and Hamburger, 2001). To determine if overexpression of PAK1 could increase Thr phosphorylation of Ebp1, we transfected MCF-7 GFP-*ebp1* cells with wild-type or constitutively active PAK1 (T423E) and tested Thr phosphorylation of Ebp1. These data indicated that Ebp1 Thr phosphorylation was enhanced after transfection of constitutively activated PAK1 (Figure 3B).

### ErbB3 binding protein Ebp1 interacts with PAK1 *in vitro* and *in vivo*

**Figure 4:**
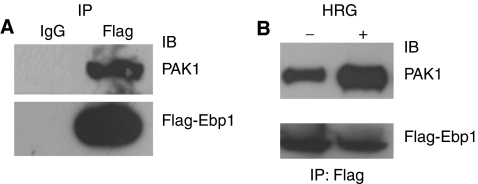
P21-activated kinase 1 and Ebp1 interact *in vivo*. (**A**) MCF-7 cells were transfected with Flag-*ebp1* and a wild-type PAK1 expression construct. Cell lysates were immunoprecipitated with a Flag antibody and blots probed for PAK1 or Flag-tagged Ebp1 as indicated. Representative to three independent experiments. (**B**) MCF-7 cells transfected with Flag-tagged *ebp1* were treated with HRG (20 ng ml^−1^) for 10 min (+) or left untreated (−). Cell lysates were immunoprecipitated with the Flag antibody and immunoprecipitated proteins probed for PAK1 or Flag-Ebp1 as indicated. Representative of three independent experiments.

We next tested the interaction of Ebp1 and PAK1 in MCF-7 cells. MCF-7 cells were transfected with a Flag-tagged Ebp1 and a PAK1 expression vector. ErbB3 binding protein was immunoprecipitated with an anti-Flag antibody and immunoprecipitated proteins probed for the presence of Ebp1 and PAK1. We found that both PAK1 and Ebp1 were immunoprecipitated with the Flag antibody (Figure 4A).

As the activity of both PAK1 and Ebp1 is modulated by HRG, we determined the effect of HRG treatment on PAK1–Ebp1 interactions. MCF-7 cells expressing Flag-tagged Ebp1 were treated with 20 ng ml^−1^ HRG for 10 min. Cell lysates were immunoprecipitated using Flag-agarose beads and immunoprecipitated proteins probed for PAK1 and Ebp1. We found that the binding of Ebp1 to PAK1 was increased in response to HRG treatment (Figure 4B).

### P21-activated kinase 1 regulation of Ebp1 corepressor functions

**Figure 5:**
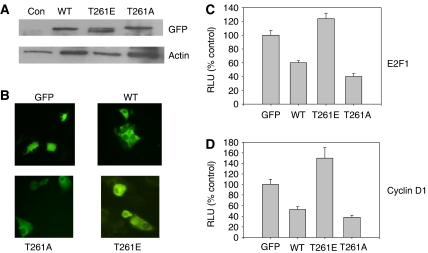
Effect of mutations at T261 on Ebp1's ability to repress transcription. (**A**) MCF-7 cells were transfected with a GFP vector control (Con) or plasmids expressing wild-type GFP-Ebp1, Ebp1 T261E or Ebp1 T261A mutants. Cell lysates were resolved by SDS–PAGE and analysed by western blotting with antibodies for GFP or actin as indicated. (**B**) Localisation of Ebp1 T261 mutants, MCF-7 cells were stably transfected with plasmids expressing GFP, wild-type GFP-Ebp1 and GFP-Ebp1 T261A or T261E mutants. The GFP tag was visualised by fluorescence microscopy in live cells. (**C**, **D**) Mutation at Thr 261 affects the ability of Ebp1 to repress transcription. MCF-7 cells were transfected with E2F1 or Cyclin D1 promoter luciferase reporter constructs, pRL-TK and GFP, GFP wild-type-Ebp1, GFP-Ebp1 T261E or GFP T261A expression plasmids. After 48 h, cells were lysed and relative luciferase units were determined as described in the Material and methods. The data are expressed as relative light units (RLU), which are the ratio of E2F1-luc or Cyclin D1-luc RLU: pRL-TK RLU for each sample. Each bar represents the mean±s.d. of eight wells. The figure is representative of three independent experiments.

To examine the possibility that the PAK1 phosphorylation site plays a role in Ebp1 function, we created T261A and T261E mutants in the context of full-length Ebp1 for use in functional assays. The data in Figure 5A demonstrate that the GFP mutants were expressed at approximately equal levels. ErbB3 binding protein has previously been reported to be primarily localised in the nucleolus and cytoplasm, with weak nucleoplasmic staining (Xia *et al*, 2001; Squatrito *et al*, 2004; Ahn *et al*, 2006). The subcellular localisation of Ebp1 was not altered by mutation of T261 as demonstrated by immunofluorescence microscopy (Figure 5B).

Transcription of both endogenous and exogenous E2F1-regulated genes is repressed by Ebp1 (Zhang *et al*, 2003). We therefore next investigated the effect of the PAK1 site mutations on the ability of Ebp1 to repress activity of luciferase reporters controlled by the E2F1 and Cyclin D1 promoters. MCF-7 cells were transfected with pRL-TK, an E2F1-Luc reporter (−225 to +1) or a Cyclin D1 reporter (1–163) and with GFP, GFP-Ebp1, GFP-Ebp1 T261E or GFP-Ebp1 T261A. Forty-eight hours after transfection, promoter activity was determined using the dual luciferase reporter assay. Wild-type *ebp1* inhibited transcription of both the E2F1 and Cyclin D1 genes 50% as reported previously (Zhang *et al*, 2003). Mutation of T261 to E, mimicking a phosphorylated state, completely abrogated the ability of Ebp1 to repress activity of both the E2F1 and Cyclin D1 promoters (Figure 5C and D). In fact, luciferase activity was significantly stimulated by the T261E mutant (*P*<0.05). The T261A mutant was more effective at inhibiting promoter activity in both cases than wild-type *ebp1* (*P*<0.05).

### Mutation at T261 affects the ability of Ebp1 to inhibit cell growth

**Figure 6:**
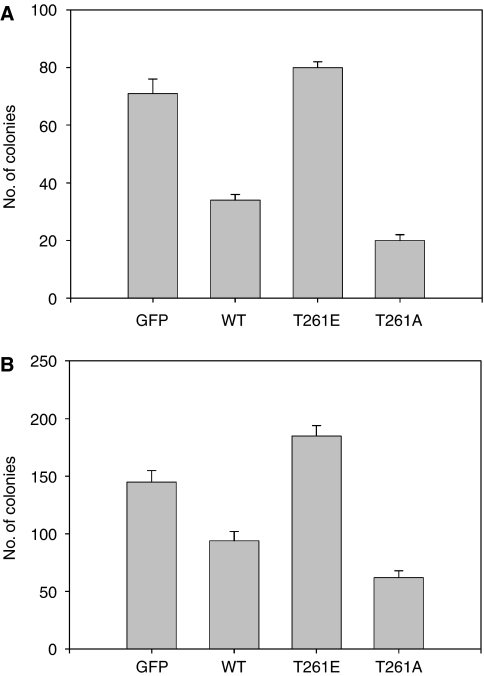
Effect of Ebp1 mutated at T261E on colony formation. MCF-7 (**A**) or AU565 (**B**) cells were transfected with plasmids expressing GFP, GFP-Ebp1, GFP-Ebp1 T261E or GFP-Ebp1 T261A as described in the Materials and methods. Cells were selected with G418 for 3 weeks. Surviving colonies were then fixed and stained with crystal violet and counted. Each bar represents mean±s.d. of six wells. The Figure is representative of three independent experiments.

Ectopic expression of Ebp1 in breast cancer cell lines inhibits colony formation (Lessor *et al*, 2000). We therefore performed a colony-forming assay to determine the effects of the PAK1 site mutation on Ebp1's ability to inhibit cell growth. MCF-7(ER+) and AU565(ER−) cells were transfected with GFP, GFP-Ebp1, GFP-Ebp1 T261E or GFP-Ebp1 T261A and selected for 3 weeks with G418. The surviving colonies were then stained and counted. GFP-Ebp1 inhibited colony formation of both cell lines as reported previously (Lessor *et al*, 2000). In contrast, the T261E mutant was completely unable to inhibit colony formation. GFP-Ebp1 T261A decreased colony growth to a greater extent than wild-type Ebp1 (*P*<0.05) (Figure 6A and B).

### Functional Ebp1 is required for tamoxifen sensitivity

**Figure 7:**
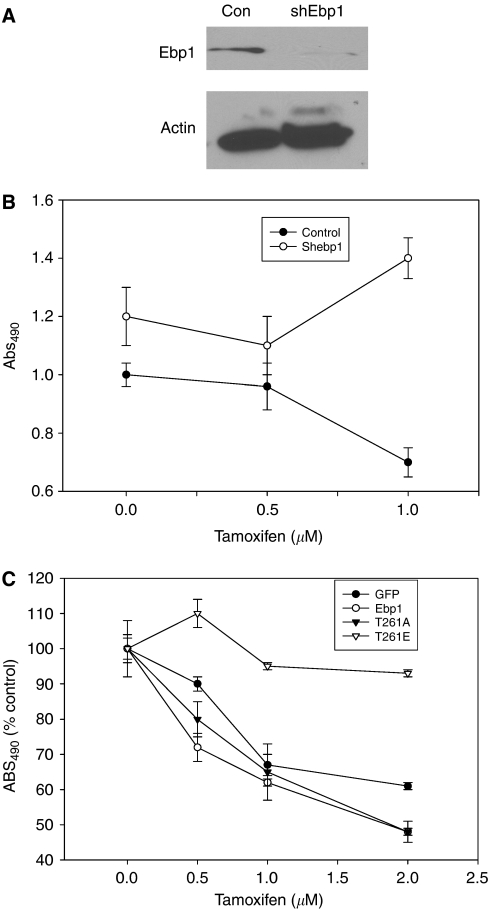
ErbB3 binding protein contributes to tamoxifen sensitivity. (**A**) Lysates of MCF-7 cells stably transduced with a control lentivirus (Con) or an Ebp1-targeted shRNA (sh Ebp1) were collected and resolved by SDS–PAGE. Western blots were probed for endogenous Ebp1 or actin as indicated. (**B**) MCF-7 cells stably transduced with a control lentivirus (control) or an shRNA *ebp1*-targeted lentivirus were incubated for 4 days in the presence of tamoxifen at the indicated concentrations or vehicle control. Viable cells were quantified using a Promega Cell Proliferation assay. Each data point represents the mean±s.d. of six wells. Similar results were observed in two independent experiments. (**C**) Sensitivity of MCF-7 cell stably transfected with T261 mutants to tamoxifen. MCF-7 cells stably transfected with GFP, GFP-Ebp1, GFP-Ebp1 T261A or T261E were plated in the presence of the indicated concentrations of tamoxifen and growth assessed by a Promega Proliferation assay 4 days later. Each data point represents the mean±s.d. of six wells. Similar results were observed in two independent experiments.

P21-activated kinase 1 has been reported to inhibit tamoxifen action in MCF-7 cells via activation of ER*α* through phosphorylation of Ser 305 (Rayala *et al*, 2006). We postulated that inactivation of Ebp1 by PAK1 may also contribute to tamoxifen resistance in hormone-dependent cells. We first tested the contribution of Ebp1 to tamoxifen sensitivity by knockdown of endogenous Ebp1 expression. As shown in Figure 7A, transduction of MCF-7 cells with an shRNA vector targeted to *ebp1* reduced Ebp1 expression compared to a lentiviral control. MCF-7 vector control cells were inhibited by tamoxifen treatment at 1 *μ*M as expected (Jhabvala-Romero *et al*, 2003). In contrast, growth of the Ebp1 knockdown cells was significantly increased at the highest concentration of tamoxifen tested (Figure 7B).

We next tested if the T261E mutant could function as a dominant negative to inhibit the ability of wild-type Ebp1 to contribute to tamoxifen sensitivity. Therefore, the sensitivity of MCF-7 cells, stably transfected with wild-type *ebp1* or the T261A or T261E mutants, to tamoxifen was examined. We found that MCF-7 cells stably transfected with the T261E mutant was no longer sensitive to tamoxifen (Figure 7C).

## DISCUSSION

A role for the PAK (p21-activated kinase) serine/threonine kinases in regulation of growth of breast cancer cells both in pre-clinical models and in patients is emerging (Kumar *et al*, 2006). P21-activated kinases are activated via a variety of extracellular stimuli and transduce their signals through multiple binding partners. Known PAK1 effectors in breast cancer pathogenesis include proteins involved in actin reorganisation, metabolic regulation, apoptosis, differentiation and transcriptional regulation (Kumar *et al*, 2006). We report here that Ebp1, a protein that binds ErbB3 and acts as a transcriptional corepressor, is a PAK1 substrate. Mutation of the Ebp1 PAK1 phosphorylation site results in inactivation of the ability of Ebp1 to repress transcription of cell cycle-regulated genes and inhibit breast cancer cell growth.

As PAK1 is activated through stimulation of cells with the ErbB3 receptor ligand, HRG (Adam *et al*, 1998), we initially hypothesised that Ebp1 could be a PAK1 substrate. Scanning the Ebp1 sequence yielded four putative PAK1 phosphorylation sites at S252, T261, S335 and S375 possessing the PAK1 motif (K/R-K/X-X-S/T). We initially found that GST-Ebp1, aa 133–306, which contains two of these sites, was phosphorylated by PAK1 in an *in vitro* kinase assay. In addition, PAK1 associated with Ebp1 in MCF-7 cells and this association was increased by HRG treatment, further supporting the relevance of the *in vitro* phosphorylation.

We, therefore, created single- and multisite alanine mutations of these predicted phosphorylation sites in GST-Ebp1 for use in a PAK1 kinase assay. Mutation of all of the putative Ser phosphorylation sites failed to alter the ability of PAK1 to phosphorylate Ebp1. However, we found that mutation of Thr 261 to Ala abrogated the ability of PAK1 to phosphorylate Ebp1. Therefore, we studied the functional significance of Thr 261 *in vivo*. Mutation of the Thr 261 site to Ala or Glu did not alter the subcellular distribution of Ebp1 in MCF-7 cells. However, mutation of Thr 261 to Ala completely abrogated the ability of Ebp1 to inhibit transcription of Cyclin D1 and Cyclin E genes in reporter assays.

P21-activated kinase 1 has previously been shown to alter the activities of other transcriptional repressors. Thus, data reported here strengthen the role of PAK1 in corepressor regulation. P21-activated kinase 1 inactivates the transcriptional corepressor CtBP, due to the changes in its cellular localisation (Barnes *et al*, 2003a). Conversely, the activity of the Notch pathway repressor SHARP is enhanced by PAK1 phosphorylation. The mechanism of the inhibition of Ebp1 transcriptional repression by PAK1 is not known. Unlike PAK1 phosphorylated CtBP, the subcellular localisation of Ebp1 mutated at the PAK1 phosphorylation site was unchanged from that of wild type. The transcriptional repression domain of Ebp1 has been mapped to the last 72 amino acids of the C terminal domain (322–394) (Xia *et al*, 2001), but it is possible that phosphorylation at Thr 261 results in changes in the three dimensional structure of Ebp1. The crystal structure of Ebp1 has recently been solved (Monie *et al*, 2007). These studies suggest that as Thr 261 is located at the beginning of an *α*-helix, its mutation may destabilise the helix.

In addition, Thr 261 phosphorylation may inhibit the interaction of Ebp1 with other transcriptional corepressors. For example, mutation of Ser 363 to Ala abrogates Ebp1 binding to the Sin3A and HDAC2 transcriptional corepressors (Akinmade *et al*, 2007). However, mutation at Thr 261 to either Ala or Glu did not prevent binding of Ebp1 to Sin3A or HDAC2 (data not shown). Thr 261 is located in an amphipathic domain predicted to interact with DNA and protein. Therefore, it is possible that a mutation at this site interrupts the interaction of Ebp1 with as yet unidentified proteins important in transcriptional repression.

Alternatively, we have shown that Ebp1 can bind to the E2F1 consensus element of endogenous promoters in a complex with Sin3A and HDAC2. It is possible that Ebp1–PAK1 interaction recruits PAK1 to a corepressor complex on E2F1 binding sites within chromatin, where PAK1 may phosphorylate and activate transcriptional repressors. In addition, we have not yet examined how mutation of Thr 261 affects the ability of Ebp1 to bind at E2F1-regulated promoters.

Overexpression of Ebp1 inhibits growth of both ER-positive and ER-negative breast cancer cells (Lessor *et al*, 2000). The T261E mutant was unable to inhibit growth of either ER-positive or ER-negative cell lines, in keeping with its inability to repress activity of Cyclin D1 and E2F1 promoters. The fact that this effect was observed in both ER-positive and ER-negative cell lines suggests that the effects of Ebp1 are not mediated via the ER. We do not yet know if the failure of mutant Ebp1 to inhibit overall cell growth is due to changes in its ability to affect the rate of cell division or apoptosis or a combination of the two. P21-activated kinase 1 has previously been shown to contribute to breast cancer cell growth via its phosphorylation of ER*α* at Ser 305, leading to ligand-independent growth. Our findings suggest that the phosphorylation and inactivation of Ebp1 by PAK1, with subsequent abrogation of Ebp1's growth inhibitory effects, is a new mechanism whereby PAK1 induces growth of breast cancer cells.

We have previously found that Ebp1 is phosphorylated and activated by HRG treatment. Thus, it appears paradoxical that the T261E mutant is inactive. It is possible that in cells, which weakly express PAK1 (such as MCF-7) (Rayala *et al*, 2006), T261 phosphorylation is low and Ebp1 remains active. In the face of high levels of PAK1, as is observed in tamoxifen resistant cells, Ebp1 becomes heavily phosphorylated, inactivating its function. This leads to the inability of Ebp1 to repress activity of E2F1-regulated cell cycle genes and inhibit cell growth. Thus, inactivation of Ebp1 may play a role in the ability of PAK1 to contribute to breast cancer progression and tamoxifen resistance.

In summary, the results reported here reveal that Ebp1 is a new substrate for the PAK1 kinase. In addition, our studies suggest that tamoxifen resistance induced by PAK1 overexpression may be related to its ability to inhibit Ebp1 function, in addition to its ligand-independent activation of ER*α*.
